# Mainz Emergency Evaluation Scoring in combination with capnometry predicts outcome in trauma patients

**DOI:** 10.1186/cc10992

**Published:** 2012-03-20

**Authors:** EH Hajdinjak, ŠG Grmec, MK Križmarić, ET Torkar, DB Buić-Rerečić, MZ Zelinka, MŠ Škufca

**Affiliations:** 1 Center for Emergency Medicine, University of Ljubljana, University of Maribor, Maribor, Slovenia; 2 Community Health Centre Ljubljana, University of Ljubljana, University of Maribor, Ljubljana, Slovenia; 3 Faculty of Medicine, University of Maribor, Slovenia

## Introduction

This prospective study assessed the efficacy of the predicting power for mortality of two different prehospital scoring systems in trauma patients. We present an improved Mainz Emergency Evaluation Scoring (MEES) in combination with capnometry (MEESc). MEESc is a new scoring system. We compared the prognostic role of outcome of these two prehospital descriptive scoring systems with the prognostic scoring system APACHE II.

## Methods

In a prehospital setting, the values of MEES and capnometry (initial and final) were collected from each patient. We added final values of petCO_2 _to the MEES scoring system and ranked from 0 to 2 so that the final maximum sum of the scoring system would be 30 without any change in the minimal score being 10. This study was performed over 10 years (from January 2000 to July 2010) and included 231 consecutive patients hospitalized for major trauma, requiring intubation at the roadside and in whom prehospital petCO_2 _has been recorded. Patients younger than 16 years and those with severe hypothermia were excluded from the study. There were 156 males and 75 females, age range 16 to 84, mean 43.6 ± 17.8 years. In hospital we calculated the APACHE II scoring system for each patient. For every scoring system, the sensitivity, specificity, correct outcome prediction and area under the ROC curve were determined.

## Results

For prediction of mortality, the best cut-off points were 19 for MEES and 22 for MEESc. The area under the ROC curve was 0.63 for MEES, 0.81 for MEESc (*P *= 0.02 vs. MEES) and 0.84 for APACHE II (*P *< 0.01 vs. MEES).

## Conclusion

There were significant differences between MEES and MEESc. MEESc improved the results of MEES in predicting outcome for severe trauma patients. The prehospital use of the improved MEESc could be an efficient communication protocol between the prehospital and hospital settings (MEESc is comparable with APACHE II).

